# Pure White Cell Aplasia and Necrotizing Myositis

**DOI:** 10.1155/2016/4161679

**Published:** 2016-03-17

**Authors:** Peter Geon Kim, Joome Suh, Max W. Adelman, Kwadwo Oduro, Erik Williams, Andrew M. Brunner, David J. Kuter

**Affiliations:** ^1^Department of Medicine, Massachusetts General Hospital, Boston, MA 02114, USA; ^2^Department of Pathology, Massachusetts General Hospital, Boston, MA 02114, USA; ^3^Department of Hematology/Oncology, Massachusetts General Hospital, Boston, MA 02114, USA

## Abstract

Pure white cell aplasia (PWCA) is a rare hematologic disorder characterized by the absence of neutrophil lineages in the bone marrow with intact megakaryopoiesis and erythropoiesis. PWCA has been associated with autoimmune, drug-induced, and viral exposures. Here, we report a case of a 74-year-old female who presented with severe proximal weakness without pain and was found to have PWCA with nonspecific inflammatory necrotizing myositis and acute liver injury on biopsies. These findings were associated with a recent course of azithromycin and her daily use of a statin. Myositis improved on prednisone but PWCA persisted. With intravenous immunoglobulin and granulocyte-colony stimulating factor therapies, her symptoms and neutrophil counts improved and were sustained for months.

## 1. Introduction

Pure white cell aplasia (PWCA) is a rare condition characterized by agranulocytosis with absent myeloid precursors in the bone marrow but preserved erythropoiesis and megakaryopoiesis. PWCA has been associated with autoimmune conditions, medications, and viral infections. Although the etiology is unknown, one potential mechanism is through immunoglobulin-mediated suppression of granulopoiesis [1–3]. Autoimmune disorders and conditions that have been associated with PWCA include autoimmune thyroiditis, type 1 diabetes, Goodpasture syndrome, primary biliary cirrhosis, and thymomas [4–7].

Other etiologies of PWCA also include medications [8–11], and viral infections [12, 13], which are likely mediated through similar immunologic mechanisms. Treatment varies but is centered on immunosuppression and removal of any offending agents [2, 6, 14]. We report a case of PWCA associated with necrotizing myositis and acute liver injury that occurred in the setting of recent exposure to azithromycin.

## 2. Case Report

A 74-year-old woman presented to our institution with a complaint of subacute bilateral proximal extremity weakness. Two weeks prior to admission, she was diagnosed with a sinus infection and received a 5-day course of azithromycin. Five days following treatment, she developed progressive painless bilateral proximal weakness over the span of two weeks involving the proximal legs, shoulders, and arms, for which she presented to the hospital.

Her past medical history included hypertension, hypothyroidism, hyperlipidemia, and a distant history of transient ischemic attacks for which she was taking atorvastatin 40 mg daily. Her medications included daily amlodipine 10 mg, losartan 100 mg, carvedilol 25 mg, triamterene/hydrochlorothiazide (37.5 mg/25 mg), aspirin 325 mg, sertraline 50 mg, vitamin D/calcium (1500 mg/2000 IU), lansoprazole 30 mg, levothyroxine 125 mcg, and twice daily alprazolam 0.25 mg as needed. She had no recent medication changes. Her family history was notable for a sibling with limited scleroderma. Physical examination on the day of admission showed a blood pressure of 155/61, temperature of 97.6°F, and inability to lift proximal legs against gravity or ambulate.

Admission laboratories showed a hemoglobin of 12.1 g/dL, white blood cell count of 4.34 × 10^3^/*μ*L (50.3% neutrophils, 35.5% lymphocytes, 8.5% monocytes, 4.1% eosinophils, and 1.4% basophils), and platelets of 165 × 10^3^/*μ*L. Chemistries were notable for blood urea nitrogen of 44 mg/dL and creatinine of 1.34 mg/dL. Liver chemistries showed alanine aminotransferase (ALT) of 545 IU/L (normal 7–33 IU/L), aspartate aminotransferase (AST) of 975 IU/L (normal 9–32 IU/L), alkaline phosphatase of 621 IU/L (normal 30–100 IU/L), direct bilirubin of 3.5 mg/dL, total bilirubin 5.0 mg/dL, total protein 7.5 mg/dL, and albumin 3.0 mg/dL. Prothrombin and partial thromboplastin times were normal. Her creatine phosphokinase (CPK) was elevated at 2597 IU/L (normal 40–150 IU/L) with an elevated aldolase level of 20.8 IU/L (normal 0–7.7). She was euthyroid on her thyroid supplementation. Immunoglobulin (Ig) levels were elevated (IgG, 1661 mg/dL; IgA, 771 mg/dL; IgM, 456 mg/dL); serum protein electrophoresis showed a normal pattern with mild diffuse increase in gamma globulin. Erythrocyte sedimentation rate was elevated at 98 mm/hr (normal 0–20 mm/hr) and C-reactive protein was also elevated at 19.4 mg/L (normal < 8 mg/L).

**Figure 1:**
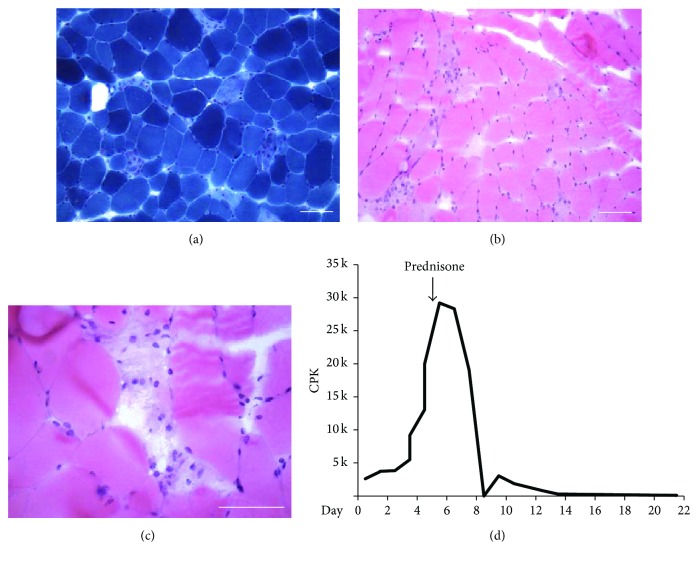
Necrotizing myositis from biopsy of the left quadriceps muscle. (a) Gomori trichrome stain showing pale focal necrosis without evidence of ragged red fibers, nemaline rods, inclusions, or increased interstitial fibrosis. 20x objective. Scale bar 100 *μ*m. (b) Haematoxylin and eosin stain showing focal necrosis without abundance of inflammatory hematopoietic cells. 200x. Scale bar 100 *μ*m. (c) Highlight of area of focal necrosis in haematoxylin and eosin. 400x. Scale bar 100 *μ*m. (d) Time-course of CPK elevation during hospitalization. Day 0 indicates day of admission.

The patient underwent extensive testing for inflammatory myopathies, autoimmune conditions, and neuromuscular diseases including myasthenia gravis. Anti-nuclear antibody was 1 : 80. Anti-La/SSB was negative but anti-Ro/SSA was elevated to 27.52 IU/mL (normal range 0–19.99 IU/mL). Myositis specific antibodies including anti-synthetase syndrome-associated (Jo-1, PL7, PL12, EJ, OJ), necrotizing myopathy-associated (SRP), statin-related autoimmune myopathy-associated (HMGCR), dermatomyositis/polymyositis-associated (Mi-2, PM/Scl-100), and inflammatory myopathy-associated (Ku) antibodies were negative [15–17]. Anti-striated muscle antibody and acetylcholine-receptor binding antibody were negative [18, 19]. Computed tomography (CT) imaging of the chest did not reveal a thymoma. Electromyography was nondiagnostic. Muscle biopsy revealed focally necrotizing myopathy with chronic inflammation consistent with an immune-mediated process; there were no characteristics of inflammatory myopathies such as polymyositis, dermatomyositis, or inclusion-body myositis (Figures 1(a)–1(c)). The biopsy also excluded mitochondrial disorders, genetic muscular disorders such as nemaline myopathy, glycogen storage disorders, connective-tissue disorders, and vasculitis. Ultrasound of the liver was unrevealing. Autoimmune hepatitis specific antibodies including anti-smooth muscle antibody, anti-LKM-1, LC1, and SLA/LP were negative. Liver biopsy was performed, revealing mild mixed portal inflammation without significant interface activity, consistent with recent liver injury either drug-induced or from systemic illness.

The patient was treated with intravenous fluids for acute kidney injury. Prednisone 80 mg daily was started on day 5 of admission for her inflammatory myopathy of unclear etiology. Her CPK peaked at 29210 IU/L on day 5 of admission before trending down (Figure 1(d)). Her strength returned towards the end of her hospitalization. Atorvastatin was held on admission; however, her AST and ALT continued to rise. Her ALT peaked at 885 IU/L on day 7 and AST peaked at 1823 IU/L on day 5 of admission. Alkaline phosphatase and bilirubin both declined over the course of her admission. The liver enzymes normalized after the initiation of prednisone.

**Figure 2:**
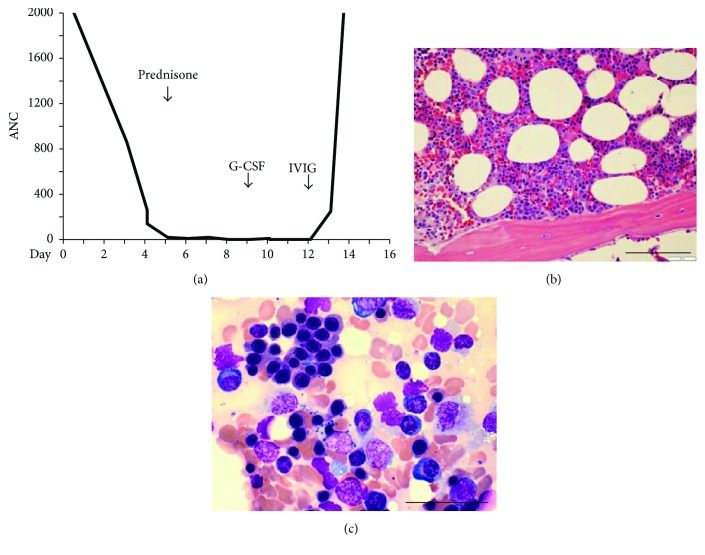
Agranulocytosis with normal erythropoiesis and megakaryopoiesis. (a) Time-course of ANC during the admission showing severe neutropenia despite initiation of prednisone. Recovery of ANC to levels >2000 occurred after initiation of G-CSF and IVIG. (b) Bone marrow core biopsy showing paratrabecular region devoid of myeloid precursors but filled with erythroid precursors and occasional megakaryocytes. 400x. Scale bar 100 *μ*m. (c) Bone marrow aspirate devoid of myeloid cells. 1000x. Scale bar 50 *μ*m.

On admission, the patient had a normal absolute neutrophil count (ANC) but on day 3 became neutropenic with ANC of 860. Other lineages were unaffected. The ANC further declined to a nadir of 0 on day 8 of admission and persisted despite prednisone 80 mg daily (Figure 2(a)). Further testing for serum hepatitis A, serum hepatitis B, serum hepatitis C, Lyme disease, Epstein-Barr virus (EBV), cytomegalovirus (CMV),* Mycoplasma*, chlamydia,* Ehrlichia*,* Anaplasma*, human immunodeficiency virus, and tuberculosis was all negative. Bone marrow biopsy revealed a normocellular marrow with normal erythroid and megakaryocytic maturation, and virtually no myeloid progenitors, consistent with agranulocytosis (Figures 2(b)-2(c)). The bone marrow differential revealed 0% myeloid blasts, 1% promyelocytes, 1% myelocytes, 0% metamyelocytes, bands, and neutrophils, 74% erythroids, 17% lymphocytes, 3% eosinophils, and 4% plasma cells. The plasma cells were polytypic based on immunochemical stains, suggesting a mild reactive plasmacytosis. No abnormal bone marrow cellular infiltrates or chromosomal abnormalities were detected by cytology, flow cytometry, and cytogenetics. CD4 : CD8 ratio was normal. Immunostains for CMV and HSV and in situ hybridization for EBV were negative, consistent with serologic findings.

Based on the biopsy, a diagnosis of PWCA was made. Treatment with granulocyte-colony stimulating factor (G-CSF) (filgrastim, 5 mcg/kg/day) was initiated (Figure 2(a)). However, no improvement in ANC was observed after 4 days of G-CSF. The patient then received one dose of intravenous immunoglobulin (1 g/kg); subsequently, her ANC improved to a peak of 9030. At 6 months from this episode, she has completed her prednisone taper with improvement in her strength, recovered her ability to walk, and has a normal ANC without additional G-CSF.

## 3. Discussion

PWCA is rare and mechanisms remain uncertain, but the disease is commonly associated with autoimmune, drug-induced, or viral causes. In our patient, autoimmune causes were considered given her family history of limited scleroderma, acute liver injury concerning for autoimmune hepatitis, and history of hypothyroidism. However, serological testing failed to identify an underlying systemic autoimmune disorder and the biopsy results were not consistent with an autoimmune condition. Therefore, it was felt that her presentation was more likely related to a drug or viral infection given her recent exposures.

PWCA from viral infection has been rarely reported. Pure red cell aplasia (PRCA) is more common after viral infections [20–23], such as in the setting of Parvovirus B19 due to an immunological response against erythropoietin or red cell precursors [24]. As in PRCA, it is plausible that a viral activation of an immune-mediated mechanism underlies PWCA. However, in this case, serum antibody testing for known infectious etiologies was unrevealing. In the absence of a viral syndrome and given the improvements in muscle and liver injury in response to prednisone, viral causes were considered less likely.

A diagnosis that potentially unifies the liver and muscle injuries with PWCA in this patient may be a drug-related immune response. The presenting symptom of muscular weakness in this otherwise healthy patient started shortly after the administration of azithromycin. Azithromycin has been associated with an increased risk of rhabdomyolysis and liver injury in patients taking recommended doses of statins [25, 26]. While this may potentially explain the patient's acute muscle and liver injury, it is less clear how this mechanism caused concurrent PWCA. Antibiotics have previously been implicated as a cause of PWCA [11]. Although no direct link between PWCA and azithromycin has been reported, one case report described azithromycin-induced agranulocytosis in an elderly patient treated for otitis media [27]. Furthermore, the sustained resolution of our patient's symptoms with immunosuppressive therapy and cessation of atorvastatin also supports the drug-related etiology as the unifying diagnosis.

A variety of immune-mediated mechanisms have been proposed for PWCA. In this patient, severe agranulocytosis initially persisted despite therapy with high-dose prednisone and started improving after the addition of IVIG. Delayed response to prednisone is possible but less likely because, in one small case series, PWCA did not improve on prednisone [28]. The improvement likely occurred with IVIG treatment, suggesting a non-T cell or natural killer cell-mediated mechanism which may respond better to IVIG, cyclosporine, or rituximab [14, 29]. One potential humoral mechanism is a neutralizing autoantibody against G-CSF. Autoantibodies against G-CSF have been reported in several cases of Felty's syndrome or systemic lupus erythematous although these patients presented with neutropenia and not necessarily PWCA [30]. Alternatively, anti-neutrophil antibodies may be induced by antibiotic exposure [31]. Such reactions could occur through molecular mimicry, a reaction in which an epitope on a drug may cross-react with self-proteins [31]. The mechanism of IVIG therapy is still under investigation but may involve immunomodulatory effects through interfering with the Fc receptor-dependent effects of these autoantibodies [32]. Complex humoral responses likely underlie mechanisms driving PWCA and future high-throughput methods may be required to yield insight into specific epitopes driving PWCA [33].
